# The influence of culture and cognitive reserve on the clinical presentation of behavioural-variant frontotemporal dementia

**DOI:** 10.1007/s00415-023-11638-w

**Published:** 2023-03-14

**Authors:** A. Skeggs, G. Wei, R. Landin-Romero, J. R. Hodges, O. Piguet, Fiona Kumfor

**Affiliations:** 1 School of Psychology, The University of Sydney, Sydney, NSW 2006 Australia; 2 Present Address: Brain and Mind Centre, The University of Sydney, 94 Mallett Street Camperdown, Sydney, NSW 2050 Australia; 3 Central Clinical School, The University of Sydney, Sydney, NSW 2006 Australia

**Keywords:** Behavioural-variant frontotemporal dementia, Culturally and linguistically diverse, Diagnosis, Presentation, Social cognition, Language

## Abstract

Characterisation of the clinical profile of behavioural-variant frontotemporal dementia (bvFTD) has predominantly been based on Western samples. Some small studies have suggested that the clinical profile may differ in culturally and linguistically diverse populations. Additionally, there is evidence that patients from non-English speaking backgrounds may have more cognitive reserve, allowing them to tolerate more disease pathology before clinical symptoms emerge. This study aims to characterise the clinical profiles of patients with bvFTD from culturally diverse backgrounds. BvFTD patients were classified as Australian-born (Australian) or Culturally and Linguistically Diverse-English-speaking (CALD-English) and Culturally and Linguistically Diverse-Language Other Than English (CALD-LOTE). Clinical features, cognitive test performance and cognitive reserve were compared between groups. Voxel-based morphometry was used to examine the neural correlates of cognitive reserve. 107 patients with bvFTD (53 Australian, 36 CALD-English, 18 CALD-LOTE) and 51 controls were included. Analysis of neuropsychiatric features revealed more elation in Australian patients compared to CALD-English patients, with trends for CALD-LOTE patients to report more irritability. CALD-LOTE patients also had higher cognitive reserve and showed relatively greater verbal than non-verbal cognitive impairment. Neuroimaging analyses revealed that higher cognitive reserve was associated with lower integrity in the frontal–temporal regions associated with typical disease pathology in bvFTD. Our findings support the hypothesis that cognitive reserve may delay early cognitive decline in culturally and linguistically diverse patients, although these patients may still show poor verbal performance due to cultural testing biases. Clinically, these results highlight the need to consider cultural and linguistic background to inform the assessment of dementia.

**Supplementary Information:**

The online version contains supplementary material available at 10.1007/s00415-023-11638-w.

## Introduction

Behavioural-variant frontotemporal dementia (bvFTD) is characterised by atrophy to the frontal and/or temporal brain regions alongside distinct changes in social behaviour and personal conduct [1]. Current consensus diagnostic criteria for bvFTD were developed based on Western samples, and only a handful of studies have investigated the clinical manifestation of bvFTD in non-Western populations [2–4]. In the United States, ~ 14% of the population is born overseas, with a similar proportion seen in the United Kingdom (14.5%) and even higher in Australia, (~ 29%) [5–7]. Despite these population-wide trends, how bvFTD presents in culturally and linguistically diverse populations has not been examined. The impact of culture may be particularly relevant for bvFTD, where the presence of diagnostic features is largely based on clinical judgement.

Two competing hypotheses have been put forward based on the limited available data. The *disease severity hypothesis* proposes that bvFTD patients from non-Western backgrounds may present with a more severe disease profile [2]. Evidence for this hypothesis comes from cross-cultural studies which compare the clinical profiles of bvFTD patients from different countries. For example, comparisons of patients from the US, Greece and Turkey found that Greek and Turkish patients had greater disease severity scores and lower cognitive scores on the Mini-Mental-State-Examination (MMSE) at presentation [8]. Similarly, a comparison between Japanese and British patients found that Japanese patients had lower MMSE scores, as well as differences in clinical symptoms (e.g., less weight gain, different changes in appetite) [9]. There is also limited evidence that non-English-speaking patients are diagnosed at a later age, potentially due to a delay in clinical diagnosis or disease onset [2, 8]. While these findings suggest cultural influences on the clinical phenotype of bvFTD, they are subject to the confounds that arise when comparing groups across multiple different sites, including inter-rater bias and procedural differences across clinicians/sites [10].

Alternatively, the *cognitive reserve hypothesis* proposes that individuals from non-English speaking backgrounds may have greater cognitive reserve, due to factors like bilingualism, which allows them to tolerate more disease pathology before exhibiting symptoms of cognitive decline [11]. Cognitive reserve accumulates over the individual’s lifespan and more accurately measures resilience to cognitive decline than static measures such as education, which only reflect a specific period of cognitive activity (e.g., early life) [12, 13]. According to this hypothesis, patients with high cognitive reserve report mild symptoms in the early disease stages of dementia, until a threshold point, where rapid cognitive decline is observed due to high neuropathological burden. In support of this reserve hypothesis, recent studies of non-English speaking (Greek, Italian) populations have found that high cognitive reserve is associated with reduced brain integrity in the frontotemporal regions associated with early disease staging in bvFTD [14, 15]. However, research testing these competing theories in bvFTD is scant and limited by comparisons across different clinical sites, where cultural effects may be confounded by procedural differences. Examination of individuals from culturally and linguistically diverse backgrounds at the same site and by the same clinicians is therefore needed to understand how bvFTD presents in typical clinical settings.

This study aimed to compare people with bvFTD who were born in Australia and were monolingual English speakers (Australian) with people diagnosed with bvFTD from culturally and linguistically diverse (CALD) backgrounds. The CALD group was divided into those who spoke English as their first language (CALD-English), and those whose first language was a Language Other Than English (CALD-LOTE). We also formally measured cognitive reserve.

## Materials and methods

### Participants

One hundred and seven bvFTD patients were recruited from FRONTIER, the younger onset dementia clinic in Sydney, Australia. All participants underwent a comprehensive examination that included a neuropsychological assessment, an MRI brain scan, and an examination by an experienced behavioural neurologist. Diagnosis of bvFTD was determined by multidisciplinary teams, including a neurologist and neuropsychologist, according to current consensus criteria [1]. In addition, 51 healthy control monolingual English-speaking Australian participants were recruited from the FRONTIER volunteer database.

**Fig. 1:**
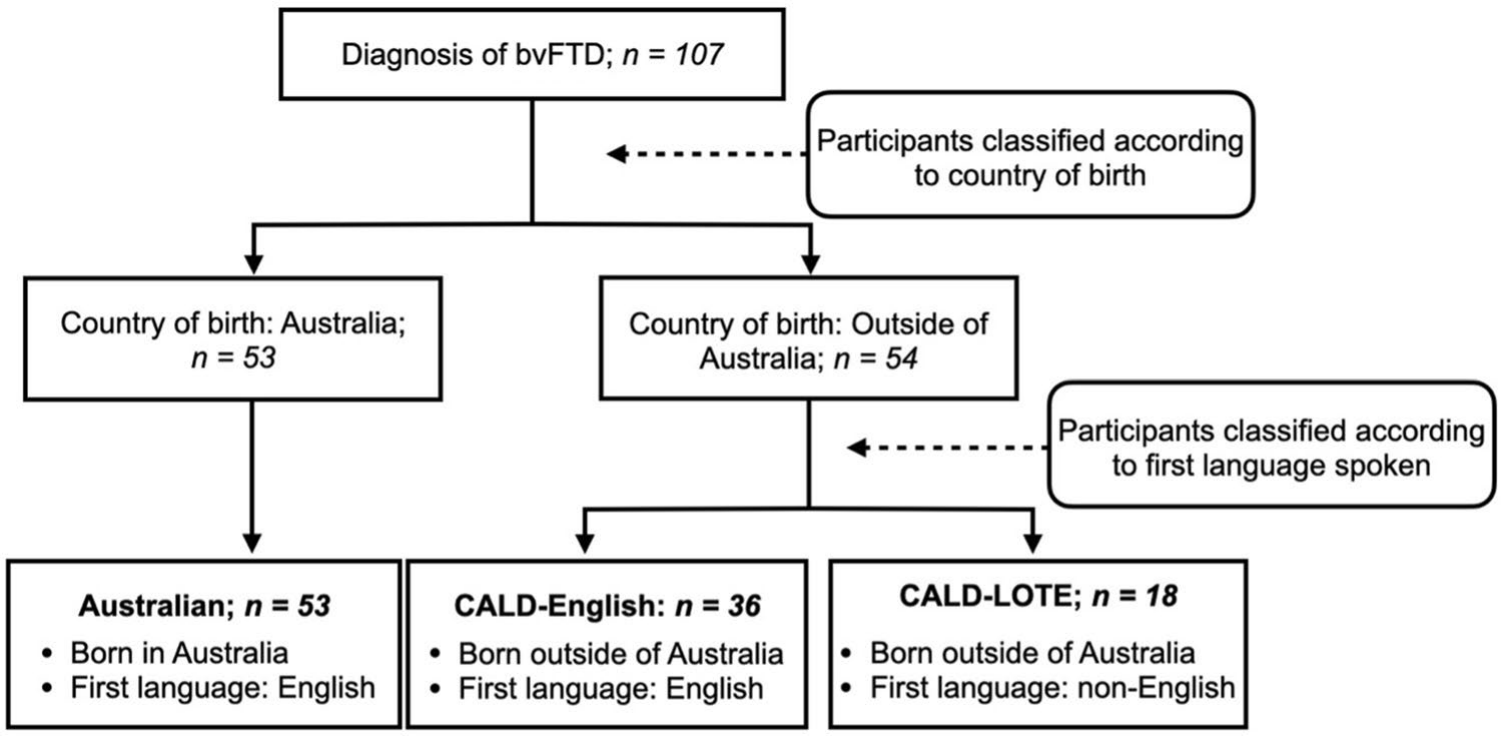
Participant classification. *bvFTD* behavioral variant frontotemporal dementia, *CALD* culturally and linguistically diverse, *CALD-LOTE* culturally and linguistically diverse-language other than English

Participants’ country of birth and languages spoken were recorded. Based on these data participants were classified into the following groups: (1) Australian born, monolingual English-speaking (Australian); (2) Culturally and linguistically diverse-English first language (CALD-English); (3) Culturally and linguistically diverse-first Language Other Than English (CALD-LOTE) (see Fig. 1). All CALD participants immigrated to Australia during their lifetime, CALD-LOTE participants were fluent in their native language and in English at the time of initial assessment. Our approach was based on previous studies that categorised participants according to country of birth and first language to isolate the influence of culture and language upon cognitive and clinical symptoms [16, 17]. All healthy controls were Australian and were selected from a larger control cohort and matched to the patient groups on sex (Table 1 for the cultural profile of each group). Exclusion criteria for all participants included: prior diagnosis of psychiatric illness or other neurological disorder, prior traumatic brain injury, history of alcohol or substance abuse, or insufficient English fluency to complete testing.

**Table 1:** Cultural profile of groups

Group	bvFTD			Healthy controls
	Australian	CALD-English	CALD-LOTE	Controls
	*n* = 53	*n* = 36	*n* = 18	*n* = 51
Country of birth	Australia (53)	British India (1) Canada (2) Fiji (1) Germany (2) Ireland (2) Netherlands (1) New Zealand (4) South Africa (3) United Kingdom (17) United States (2)	Belarus (1) Belgium (1) Bosnia (1) Brazil (1) Chile (1) Czech Republic (1) Cyprus (1) Ecuador (1) Fiji (1) Hungary (1) India (1) Italy (1) Slovak Republic (1) Sri Lanka (1) The Philippines (1) Vietnam (2) Yugoslavia (1)	Australia (51)
First language	English	English	Language other than English	English
Other languages spoken (Y:N)	0:53	8:16	18:0	0:51
Formal English schooling				
None			5	
High School	53	36	9	51

Formal English schooling refers to a number of patients who obtained formal qualifications in English

*bvFTD* behavioral variant frontotemporal dementia, *CALD* culturally and linguistically diverse, *CALD*-*LOTE* culturally and linguistically diverse-language other than English. Missing data: Formal English Schooling: CALD-LOTE: 4

### Demographic and clinical measures

Patients’ clinical and diagnostic behavioural features were systematically assessed according to current diagnostic criteria during the clinical examination and were recorded as present or absent [1]. Neuropsychiatric features were reported by carers using the Neuropsychiatric Inventory (NPI) and included aggression, agitation, delusions, hallucinations, mood disturbance, anxiety, and mental rigidity [18].

The Frontotemporal dementia Rating Scale (FRS) provided a measure of disease severity, and an associated Rasch score, with higher Rasch scores indicating less severe disease stage [19]. Disease duration was calculated as the number of years between the estimated first onset of symptoms and clinical diagnosis.

Cognitive reserve was retrospectively evaluated using the Cognitive Reserve Index Questionnaire (CRIq) [12]. The total CRIq score includes three subscores: education, working activity, leisure activities. Due to the retrospective nature of the data, only information on Education (years) and working activity (level of profession/years in the profession) was available. Therefore, a revised Cognitive Reserve Index Score was calculated as the average of patients’ education and working activity subscores.

### Cognitive measures

Participants’ general cognitive function was assessed using the Addenbrooke’s Cognitive Examination (ACE) [20]. Where relevant, ACE-R scores were transformed into ACE-III scores using validated conversion algorithms [21]. Working memory was assessed using the Digit Span backwards task [22, 23]. Trail Making Test: part A and Digit Span forwards were used to measure attention [24]. Language was assessed using the Sydney Language Battery (SYDBAT) naming and semantic association subtasks [25]. Executive function was assessed with the verbal fluency test and Trails Making Test: part B [26]. The Rey Figure Complex copy and recall task were used to assess visuospatial ability and memory, respectively [27]. Social cognition was assessed using the Facial Affect Discrimination Task (FADT) and Facial Affect Selection Task (FAST) [28, 29]

### Statistical analyses

Data were analysed using IBM SPSS (v. 26). One-way ANOVAs were conducted for continuous variables (e.g., age, education, Cognitive Reserve Index Score, age of onset/diagnosis, disease duration, FRS score) and chi-square analyses for categorical variables (e.g., sex). Further chi-square test analyses were conducted to examine group differences in the number of diagnostic features and individual diagnostic features.

Verbal and non-verbal composite scores for the cognitive tests were calculated. Specifically, test scores were converted to standardised *Z *scores for verbal tasks (ACE-III, Digit Span forwards/backwards, SYDBAT Naming) and for non-verbal tasks (Trail Making A and B tasks, RCF copy and RCF 3-min score, SYDBAT Semantic Association). *Z* scores were calculated based on the mean and standard deviation of the Control Group. These scores were averaged to create a “verbal” and “non-verbal” composite cognitive score for each participant. Social cognition scores were similarly transformed into *Z *scores and classified as “verbal” (FAST score) or “non-verbal” (FADT score). Repeated measures ANCOVAs were run to examine whether there was an interaction effect between group and test type (verbal vs. non-verbal) for cognitive and social cognition test scores. Age and education were included as covariates to account for demographic influences. Sidak corrections were applied to all post-hoc tests to account for the multiplicity of testing. Linear regression analyses were conducted with CRIq as an independent variable and verbal and non-verbal scores as dependent variables. Statistical significance was set to *p* < 0.05, unless otherwise stated.

### Neuroimaging

#### Neuroimaging acquisition

Whole-brain high-resolution T1-weighted structural magnetic resonance imaging (MRI) brain scans were available for 115 participants (81 bvFTD patients and 34 controls). Images were acquired with the following protocol: coronal orientation, matrix 256 × 256, 200 slices, 1 mm in-plane resolution, 1 mm slice thickness, echo time/repetition time: 2.6/5.8 ms, flip angle 8°. 85 scans were collected using a 3 T Phillips scanner and 30 scans were collected using a 3 T GE scanner. Sequences from both sites are equivalent and comprehensive harmonization testing was conducted to ensure compatibility.

#### Neuroimaging processing

MRI data were analysed using the FSL voxel-based morphometry (VBM) suite https://www.fmrib.ox.ac.uk/fsl/fslvbm/index.html [30–33]. Structural images were brain-extracted using BET, and then, tissue segmentation was conducted with automatic segmentation (FAST) [34]. Resulting grey matter partial volume maps were aligned to the Montreal Neurological Institute standard space (MNI52) using non-linear registration (FNIRT), which uses a *b-*spline representation of the registration warp field [35, 36]. Then, a study-specific template was created and the native grey matter images were non-linearly re-registered. The registered partial volume maps were modulated by dividing them by the Jacobian of the warp field and the modulated, segmented images were smoothed with an isotropic Gaussian kernel with a sigma of 3 mm.

#### Voxel-based morphometry analyses

Voxel-wise general linear models (GLM) were applied to investigate differences in grey matter intensities between groups via whole-brain permutation-based non-parametric testing, with 5000 permutations per contrast [37]. A conservative cluster extent threshold of 300 contiguous voxels was applied for group comparisons, with a significance threshold of *p* < 0.001, uncorrected for multiple comparisons.

To examine cognitive reserve, correlations between Cognitive Reserve Index Scores and grey matter intensity were conducted in all patient groups combined, with age, sex and disease duration included as covariates. Covariate analyses are reported at a significance of *p* < 0.01 uncorrected for multiple comparisons, with a conservative cluster extent threshold of 150 contiguous voxels. Regions yielding significance were superimposed on the MNI standard brain using mricron software (https://www.nitrc.org/projects/mricron), and anatomical labels were determined with reference to the Harvard–Oxford probabilistic cortical and subcortical atlases in FSL.

### Ethics approval

All participants provided informed consent in accordance with the Declaration of Helsinki [38]. The study was approved by the South-eastern Sydney Local Health District ethics committee and the University of Sydney ethics committees (Australia).

### Data availability

The data are not publicly available due to ethical requirements to ensure patient confidentiality and privacy. The data that support the findings of this study are available on request from the corresponding author.

## Results

### Demographics

As reported in Table 2, sex distribution was not different between groups (*p* = 0.973), although significant differences were observed for age and education (both *p* values ≤ 0.001). Control participants were older than bvFTD patients (all *p* values < 0.001). Controls and CALD-LOTE had significantly more years of education than the Australian and CALD-English groups (all *p* values < 0.05).

**Table 2:** Demographic and clinical variables across groups

	Australian	CALD-English	CALD-LOTE	Controls	Test statistic	*p*
Sex (M:F)	34:19	25:13	12:6	32:19	0.23	0.973
Age, y	58.96 ± 5.76	60.26 ± 8.74	60.88 ± 8.60	71.84 ± 6.91	32.80	< 0.001
Education,	12.06 ± 1.87	12.13 ± 2.31	13.78 ± 2.71	13.81 ± 2.15	8.03	< 0.001
Age of onset, y	58.96 ± 5.76	59.94 ± 8.75	61.18 ± 8.88	–	0.62	0.540
Age of diagnosis, y	63.26 ± 5.80	63.81 ± 9.26	65.39 ± 9.79	–	0.49	0.611
Disease duration, y	4.26 ± 2.51	4.38 ± 2.74	4.26 ± 2.93	–	0.02	0.978
FRS Rasch score	− 0.38 ± 1.24	− 0.72 ± 1.92	− 0.25 ± 1.07	–	0.75	0.473

Data are presented as mean ± standard deviation

*bvFTD* behavioral variant frontotemporal dementia, *CALD* culturally and linguistically diverse, *CALD-LOTE* culturally and linguistically diverse-language other than English, *FRS* Frontotemporal Dementia Rating Scale. FRS stage: 1.91 to − 0.40 = moderate; − 0.39 to − 2.58 = severe. Missing data: Age of Onset: 3 CALD-English; 1 CALD-LOTE, Disease Duration: 3 CALD-English; 1 CALD-LOTE, FRS logit score: 2 CALD-English; 2

No significant differences were observed for the age of onset, disease duration, age of diagnosis or dementia severity (all *p* values > 0.05). The mean age of onset (*p* = 0.540) and mean age of diagnosis (*p* = 0.611) were older in both the CALD bvFTD groups, although this difference was not statistically significant.

### Clinical profile according to cultural and language background

**Fig. 2:**
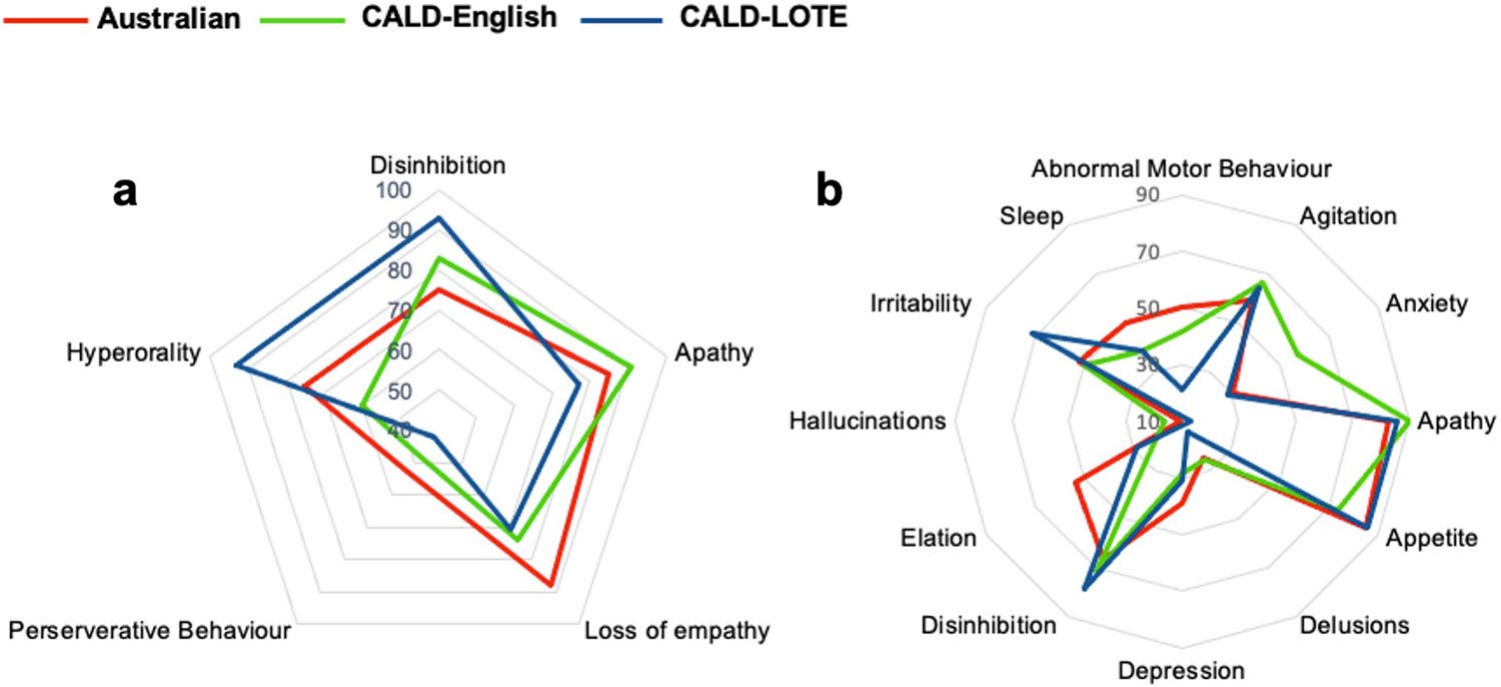
Patterns of behavioural features at initial clinical assessment. Group profiles are indicated by % of cases who fulfilled criteria at the presentation. **a** Shows the % of cases who fulfilled diagnostic features as recorded by neurologists during initial clinical assessment. **b** Shows the % of cases who fulfilled neuropsychiatric features as reported by carers on the NPI. *CALD* culturally and linguistically diverse, *CALD-LOTE* culturally and linguistically diverse-language other than English, *NPI* Neuropsychiatric Index

Examination of the presence of diagnostic features found no significant differences in the frequency of these features between groups (Fig. 2a, analyses are reported in Supplementary Table 1). However, interesting trends were noted. CALD-LOTE patients appeared to more commonly present with a disinhibited profile (92.9%) relative to CALD-English (82.6%) and Australian patients (75.0%). Conversely, Australian patients (85%) were more commonly apathetic than CALD-LOTE patients (78.6%). Hyperorality showed the greatest disparity between groups and was present in 93% of CALD-LOTE patients compared to 75% of Australian patients. Perseverative behaviour was the least common feature (< 55% in each group). The number of diagnostic criteria fulfilled at the presentation was similar across groups (${X}^{2}$= 2.60,* p* = 0.838; Supplementary Fig. 1).

Figure 2b shows the groups’ profiles on the Neuropsychiatric Inventory (analyses provided in Supplementary Table 1). Elation differed between groups, ${X}^{2}$ = 9.21, *p* = 0.010. Post hoc comparisons revealed that Australian patients showed significantly more elation (53.3%) than CALD-English patients (20.0%; *p* = 0.004). While the presence of symptoms on the other NPI subscales did not statistically differ between groups, the following trends were noted. Australian patients more commonly reported sleep disturbance (50%) than the CALD-English (38.7%) and CALD-LOTE (38.5%) groups. Anxiety tended to be more commonly reported in CALD-English patients than the other groups (56.7%), while CALD-LOTE patients more commonly reported high irritability (71.4%) than the other groups.

### Cognitive profile

**Fig. 3:**
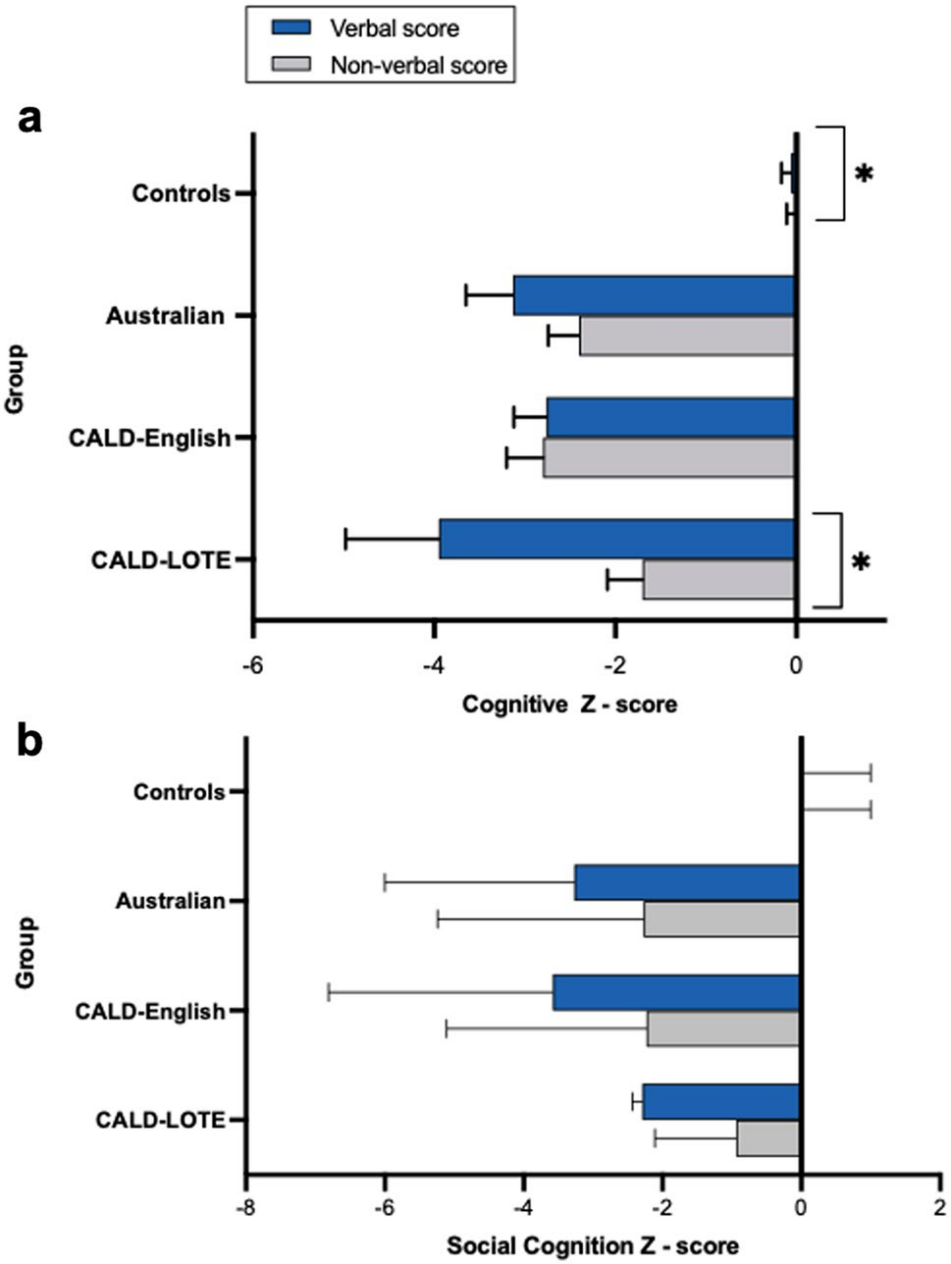
Verbal and non-verbal scores across groups. Mean verbal and non-verbal cognitive and social cognition scores for Controls, Australian, CALD-English and CALD-LOTE groups. All scores are presented as *z* scores: *Cognitive Verbal Score* composite score across verbal neuropsychological tasks, *Cognitive Non-verbal Score* composite score across non-verbal neuropsychological tasks, *Social Cognition Verbal Score* FAST score, *Social Cognition Non-verbal Score* FADT score. Error bars represent standard errors. *Significant within-group difference in verbal and non-verbal score. *CALD* culturally and linguistically diverse, *CALD*-*LOTE* culturally and linguistically diverse-language other than English, *FAST* facial affection selection task, *FADT* facial affection discrimination task

As shown in Fig. 3a, a statistically significant interaction between group and test type was observed, *F*(3,141.00) = 7.47, *p* < 0.001. Post hoc comparisons revealed significant between-group differences. As expected, all bvFTD groups performed worse than controls on both verbal and non-verbal tests (all *p* values < 0.01), with no difference between bvFTD groups. Notably, within-group comparisons revealed that the CALD-LOTE group performed significantly better on the non-verbal than the verbal composite (*p* < 0.001), whereas the CALD-English and Australian groups showed a similar level of performance on verbal and non-verbal tasks. Controls scored significantly higher on the non-verbal than the verbal composite task (*p* = 0.010), although the mean difference was small (*M* = 0.06). Groups' performance on each individual cognitive task are provided in Supplementary Table 2.

On tasks of social cognition, the interaction between group and test type was also significant, *F*(3,126) = 11.60, *p* < 0.001. Post hoc pairwise comparisons revealed that the Australian and CALD-English groups had significantly lower scores than Controls (all *p* values < 0.001) on the non-verbal affect discrimination task. In contrast, no difference was seen between CALD-LOTE patients and Controls (*p* = 0.653). On the verbal affect selection task, all bvFTD groups had significantly worse scores than Controls (all *p* values < 0.05). Within groups, post hoc comparisons revealed no significant differences in the verbal and non-verbal social cognition scores for any group (all *p* values > 0.05; Fig. 3b).

### Cognitive reserve and neuroimaging

Overall, Cognitive Reserve Index Scores differed between groups (*p* < 0.001), with Australian patients and CALD-English patients having lower cognitive reserve than Controls (both *p* values < 0.001). In contrast, the CALD-LOTE group had a similar level of cognitive reserve as Controls (*p* = 0.896). The difference in cognitive reserve was not significantly different between the bvFTD groups. Regression analyses were used to examine the effects of cognitive reserve on verbal and non-verbal test scores (summarised in Table 3). Cognitive Reserve Index Scores accounted for a significant amount of variance in a verbal cognitive score (57%), nonverbal cognitive score (33%) and verbal social cognition score (32%).

**Table 3:** The effects of cognitive reserve on cognitive and social cognition scores

Predictor	Outcome variable			
	Verbal cognitive	Nonverbal cognitive	Verbal social cognition	Non-verbal social cognition
Cognitive Reserve Index Score	*R*^2^ = 0.057 (*p* = 0.003) *t* = 3.04	*R*^2^ = 0.033 (*p* = 0.027) t = 2.24	*R*^2^ = 0.032 (*p* = 0.040) *t* = 2.08	*R*^2^ = 0.023 (ns) *t* = 1.78

Results from simple linear regression models

*t *unstandardised regression coefficient, *ns *not significant at 0.05 threshold

### Neuroimaging

**Fig. 4:**
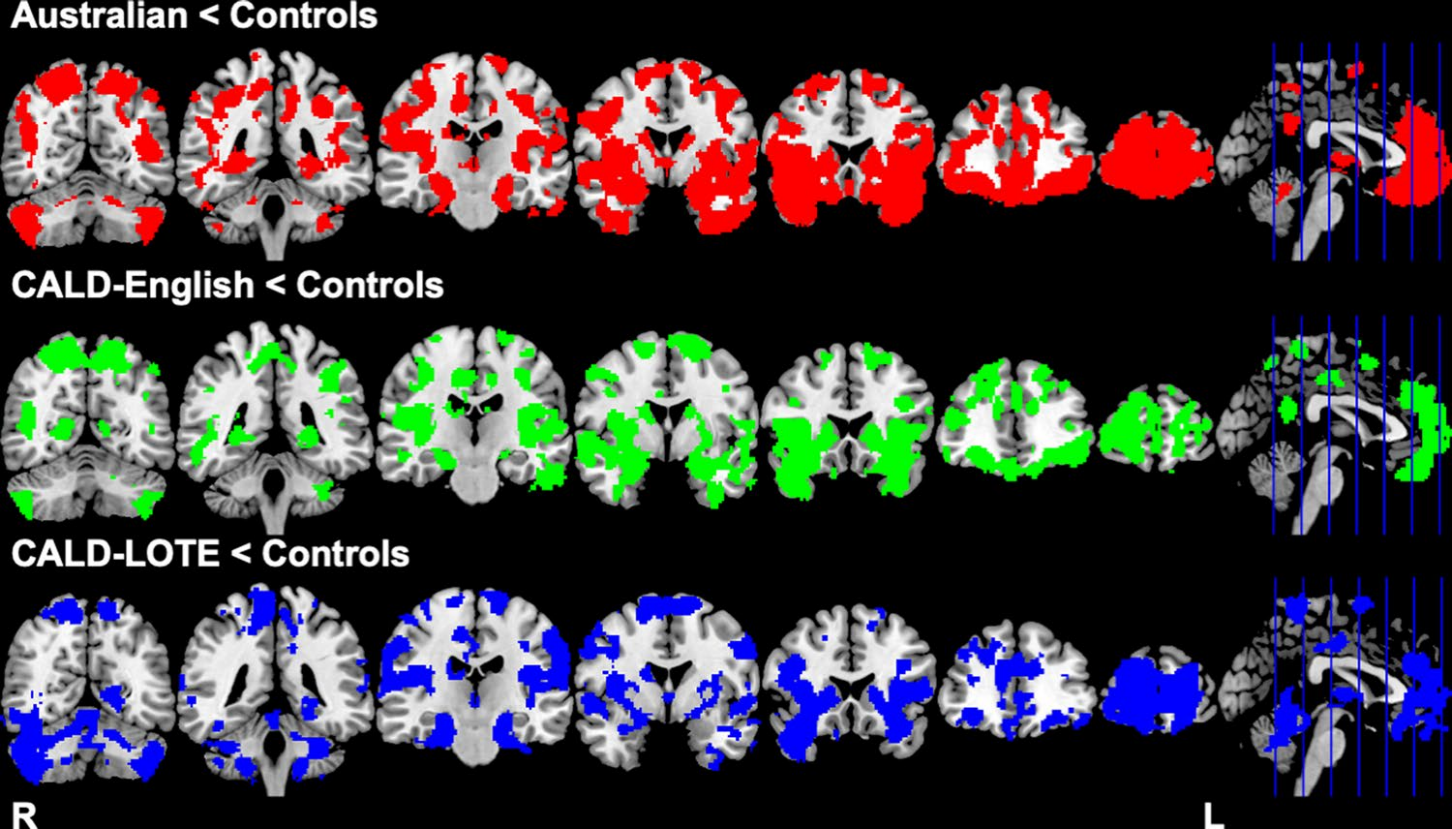
Regions of brain atrophy in bvFTD patient groups compared to controls, radiological orientation. Red-colored regions show regions of reduced grey matter intensity in Australians compared to Controls. Green-colored regions show regions of reduced grey matter intensity in CALD-English compared to HC. Blue-colored regions show regions of reduced grey matter intensity in CALD-LOTE compared to HC. Results are reported at *p* < 0.05 corrected for family-wise error with a cluster extend threshold of 300 contiguous voxels. Clusters are reported at *t* (80) > 2.37. MNI coordinates: *y* = − 60, − 40, − 20, 0, 20, 40, 60. *L* left, *R* right, *MNI* Montreal Neurological Institute, *CALD* culturally and linguistically diverse, *CALD-LOTE* culturally and linguistically diverse-language other than English

BvFTD groups showed reduced grey matter intensity compared to controls in regions in which typical patterns of atrophy are observed (Fig. 4, see also Supplementary Table 3). In brief, compared with controls, patients with bvFTD showed decreased grey matter intensity in frontal and temporal regions, including the frontal pole, orbitofrontal cortex, temporal pole, fusiform gyrus, paracingulate gyrus, as well as subcortical regions, including the hippocampus and amygdala.

**Fig. 5:**
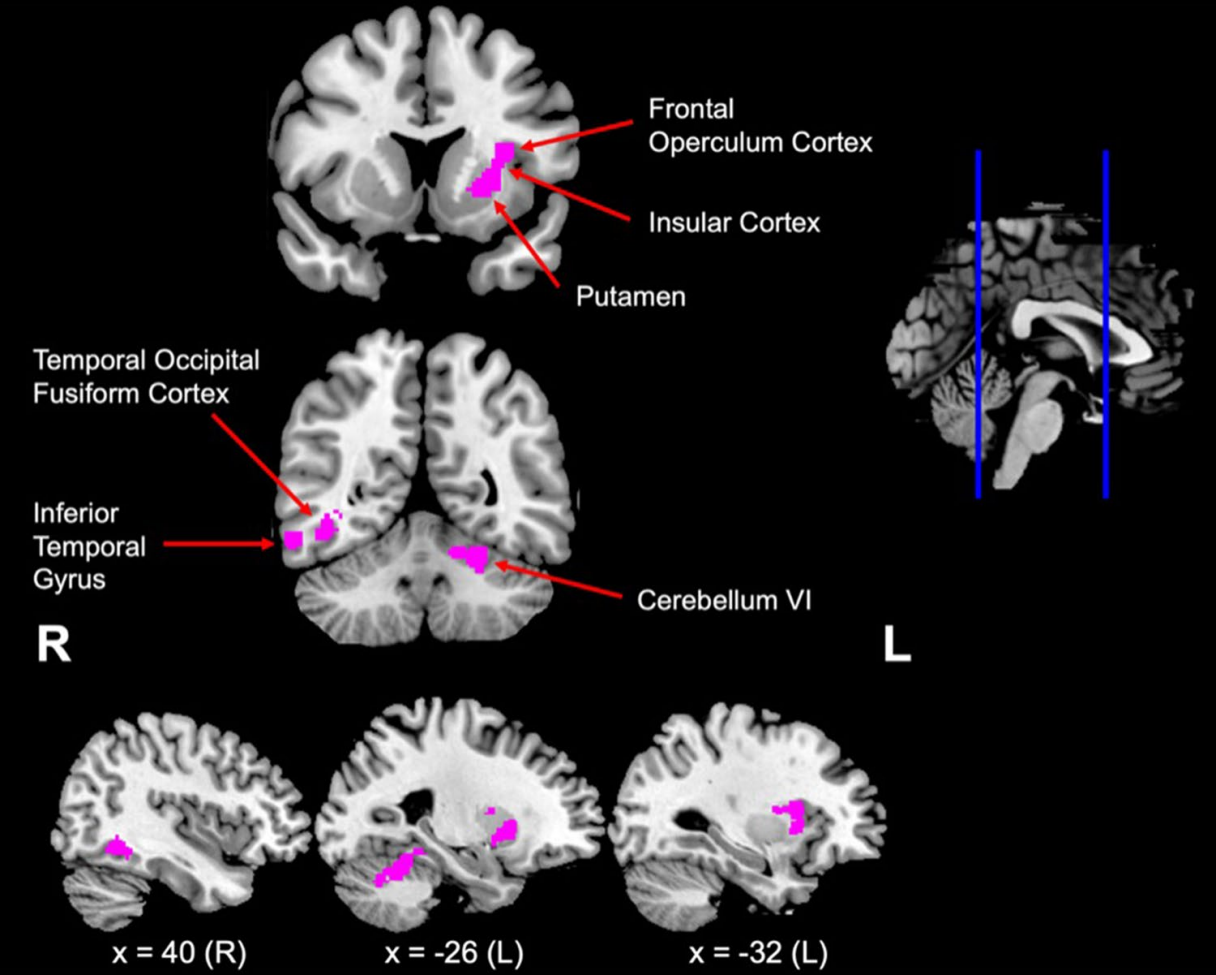
Regions of grey matter intensity reduction that covary with Cognitive Reserve Index Scores in all bvFTD patient groups, radiological orientation. The image shows regions of reduced grey matter intensity in bvFTD that covary with Cognitive Reserve Index Scores, after controlling for age, sex and disease duration. Significant clusters were thresholded at *p* < 0.01 uncorrected, with a cluster-extent threshold of 150 voxels. Clusters are reported at *t*(114) > 2.36. Arrows point to regions of significant clusters. MNI coordinates: *y* = 54, 12; *x* = 40, − 26, − 32. *L *left, *R *right, *MNI *Montreal Neurological Institute

The covariate analysis in all patients combined demonstrated that a higher Cognitive Reserve Index was associated with decreased integrity of the left frontal operculum cortex, putamen, insular cortex, right temporal occipital fusiform cortex and left cerebellum VI (Table 4, Fig. 5).

**Table 4:** Voxel-based morphometry results showing regions of grey matter intensity reduction that covary with Cognitive Reserve Index Scores in all bvFTD patient groups

Regions	Hemisphere	Number of voxels	MNI coordinates		
			*X*	*Y*	*Z*
Putamen extending to the insular cortex, frontal operculum cortex	L	301	− 32	12	14
Cerebellum (Left VI)	L	298	− 20	− 48	− 20
Temporal occipital fusiform cortex extending to the inferior temporal gyrus	R	198	40	− 54	− 6

Results uncorrected at *p* < 0.01 with a cluster extent threshold of 150 contiguous voxels, covaried for age, sex and disease duration. Clusters are reported at *t* (80) > 2.37

*L *left, *R *right, *MNI *Montreal Neurological Institute

## Discussion

How the clinical presentation of dementia varies depending on an individual’s background has been largely overlooked. Here, we examined the influence of culture (place of birth), language and cognitive reserve. We found that bvFTD patients from Australian and CALD-English-speaking backgrounds presented with similar diagnostic features, showing high apathy and loss of empathy. In contrast, CALD patients from non-English backgrounds reported greater disinhibition and hyperorality, which is in line with previous studies in Japanese and Indian cohorts [2, 9]. On cognitive testing, the CALD-LOTE group performed worse on verbal than non-verbal tasks, whereas both English-speaking bvFTD groups showed similar performance across task type. Notably, while neuroimaging analyses showed the expected pattern of brain atrophy in all three groups, individuals with higher cognitive reserve showed more brain atrophy in brain regions typically associated with bvFTD, including the insula, frontal operculum and temporal fusiform regions. Here, we discuss how these findings inform our understanding of the relationship between cultural background, cognitive reserve, and the clinical presentation of bvFTD in patients from culturally diverse backgrounds.

According to the disease severity hypothesis, people from non-Western backgrounds show more diagnostic features at presentation, have a more severe disease, and a shorter disease duration. Contrary to this hypothesis our results showed that disease severity and disease duration were not significantly different across bvFTD groups. Rather, the observed pattern of results is more closely aligned with the cognitive reserve hypothesis. Individuals who were born overseas and whose first language was not English performed similarly to Controls on the non-verbal composite score, whereas the Australian and English-speaking groups were significantly impaired across verbal and non-verbal tasks compared to controls. These results indicate that patients from CALD backgrounds have higher cognitive reserve due to demographic (e.g., education and working activity) and cultural factors (e.g., bilingualism), which leads to a relatively mild profile early in the disease course [14, 39]. Consistent with previous studies of non-English speaking bvFTD groups, high cognitive reserve was associated with greater disease burden in the insula, frontal operculum and temporal fusiform regions [14, 15]. In terms of clinical symptomology, these regions have been associated with impaired emotion recognition and perspective taking, as well as the behavioural symptoms of apathy, disinhibition, and cognitive empathy in bvFTD [40–42]. Overall, these results support the hypothesis that patients with greater cognitive reserve are able to tolerate a greater neuropathological burden before exhibiting clinical symptoms and implicate cognitive reserve in brain regions that are important for social functioning in bvFTD. In clinical assessment, high cognitive reserve may potentially mask early clinical symptoms in patients from educated/bilingual diverse backgrounds, even if these patients’ neuroimaging profiles show evidence of disease-related atrophy.

While we found limited evidence of a more severe disease profile in patients with bvFTD from CALD backgrounds, we did find evidence of differences between bvFTD groups in the patterns of neuropsychiatric features observed and the profile of performance on cognitive testing. As noted above, bvFTD patients from a non-English speaking background tended to present with a more disinhibited, irritable profile and an increased tendency towards hyperorality. A tendency towards disinhibition and hyperorality have also been reported in Indian and Japanese samples [2, 9], and may potentially reflect cultural differences in the manifestation of clinical dementia symptoms. Some evidence suggests that acculturative stress (the chronic stress of assimilating to a new culture) is related to clinical symptoms such as disinhibited eating, which may in part explain why disinhibition/hyperorality manifest as salient symptoms in dementia patients from immigrant backgrounds [43].

In contrast, English-speaking patients (from Australia and born overseas) appeared to have a profile characterised by apathy and loss of empathy. The behavioural profile of these English-speaking patients is similar to current diagnostic criteria, where apathy was the most reported symptom, while hyperorality was the least reported [1]. More apathy in Caucasian patients has previously been reported in Alzheimer’s disease whereas more irritability was observed in Hispanic patients [44]. Interestingly, symptoms may also be associated with different neural correlates. For example, apathy was correlated with less superior frontal volume in European patients, but with rostral anterior cingulate cortex volume in Hispanic patients [45]. Together, these findings suggest that clinical symptoms may be due to different neurobiological mechanisms across cultural groups [46].

Sociocultural influences on clinical profiles are also important to consider. In Western/individualistic cultures, dementia syndromes (and their associated cognitive impairment) carry negative connotations and cognitive and behavioural changes are often stigmatised in families [47]. This “negative” social reception may exacerbate individuals to disengage from their environment and heighten symptoms of apathy. In contrast, behavioural and cognitive changes are often considered normal features of aging in non-Western/collectivist cultures and symptoms such as disinhibited behaviours are less likely to be pathologized by families [2]. This acceptance may lead individuals to express disinhibited behaviours more freely, as they are not considered unusual within the cultural context. Additionally, individuals in non-Western cultures are rarely excused from their responsibilities because of cognitive decline and this expectation of continued familial/social engagement may explain why patients tend to present as less apathetic. These clinical-cultural interactions may also be amplified by heterogeneity in the way psychiatric features are understood and reported by carers from different cultural groups [4]. Understanding the mechanisms behind cultural and social influences on clinical symptoms in bvFTD remains an important area for future research, especially because current diagnostic criteria may be less valid and reliable for patients from diverse backgrounds. As cultural perceptions may also influence patient autonomy, examining cultural differences in caregiver behaviours will also be important for interventions aimed at improving functional independence.

Cognitive performance also varied depending on cultural and language background. Individuals whose first language was not English had considerably lower verbal than non-verbal scores. This difference was not observed in both English-speaking bvFTD groups. These results demonstrate that cognitive performance is impacted by verbal task demand/testing biases [48]. Studies of healthy and clinical populations consistently report that patients from non-English speaking backgrounds who are assessed in English show poorer performance than their English-speaking counterparts. Importantly, these testing biases appear even in participants with high acculturation and English proficiency, suggesting that individuals from non-English speaking backgrounds are likely to be disadvantaged on verbal tests irrespective of their English language fluency [17]. Our results demonstrate the importance of including non-verbal tasks (e.g., Rey Complex Figure Task) which are less susceptible to cultural biases and language confounds [48, 49], especially when assessing patients from diverse backgrounds.

We observed a similar pattern of results on the social cognition tasks, where patients whose first language was not English showed impairment on the verbal task but not the non-verbal task. This pattern suggests that, although the verbal demand of this task is limited, it may nevertheless be subject to testing biases. The affect selection task requires patients to identify verbally presented emotional stimuli (e.g., Identify the sad face), a process that bilingual patients may find more difficult in their second language [50, 51]. Tests of social cognition that are validated in culturally and linguistically diverse groups are lacking, and represent an important focus for future research given the potential impact on clinical diagnosis [52].

Interestingly, the cognitive profile of the patients from non-English speaking backgrounds may also be influenced by their relatively higher cognitive reserve. That is, both cognitive reserve and testing biases may influence the overall dementia profiles of patients from non-English speaking/culturally diverse backgrounds. The degree to which each of these cultural factors influences performance likely depends on the individual and the clinical context. To address this, clinicians should apply demographically adjusted norms which will improve the equitable assessment of patients from these backgrounds and reduce diagnostic errors [53]. These findings also highlight the importance of additional culturally competent practices, like the use of translators or translated assessments for patients from non-English speaking backgrounds [54]. Finally, these clinical considerations are relevant for the assessment of other neurological diseases, including Alzheimer’s disease and traumatic brain injury, where normative data for culturally diverse groups and guidelines for culturally informed practice are also lacking.

While the study’s design was able to minimise cross-cultural and procedural confounds in the results, some limitations should be noted. First, we did not collect detailed information on participants’ cultural background, and future studies should consider additional cultural factors (e.g., acculturation, immigration status) as well as demographic factors such as socioeconomic status, as these are also likely to influence test performance in culturally diverse populations [55]. Future studies which intentionally recruit a more diverse sample, particularly those who are less educated and not fluent in English, will be important to characterise the clinical profiles of patients from other cultural backgrounds. This should include samples from underrepresented racial minority groups such as Hispanic/Latino(a)s who were not represented in the present study. Importantly however, Australia’s immigration policies have historically focused on attracting skilled migrants with some level of English proficiency. Therefore, the characteristics of culturally diverse participants (i.e., educated professionals) in this study are considered representative of Australia’s broader immigration population [56]. Moreover, the number of immigrants with English fluency is projected to continue increasing over the coming decades, ensuring the relevancy of the present findings for future assessment guidelines. As we were primarily focused on patients’ clinical profiles, we did not recruit matched Control groups for the CALD groups. Future research that includes CALD Controls will be important to further explore the cognitive profiles and testing biases experienced by CALD individuals. Additionally, as we collapsed across countries and languages, our CALD groups were multicultural. We intentionally took this approach to most closely reflect clinical practice in multicultural contexts. However, future research in homogeneous cultural groups will be useful to shed light on how performance may be influenced differently across cultures (e.g., Western vs. Eastern cultural groups).

This study is the first to examine how patients’ cultural and linguistic background influences cognitive reserve and clinical presentations of bvFTD. These results support the cognitive reserve hypothesis and demonstrate how cultural and linguistic influences may lead to a degree of resilience to early cognitive decline. By identifying the clinical relevance of several cognitive and cultural factors, we highlight the importance of including diverse populations in future dementia research as a critical step to improving cultural sensitivity in clinical practice.

## Supplementary Information

Below is the link to the electronic supplementary material.

Supplementary file1 (DOCX 167 KB)

## Abbreviations

ACE-III: Addenbrooke’s Cognitive Examination-III
AD: Alzheimer’s disease
bvFTD: Behavioural-variant frontotemporal dementia
FADT: Facial Affect Discrimination Task
FAST: Facial Affect Selection Task
FRS: Frontotemporal dementia Rating Scale
VBM: Voxel-based morphometry
