# Supplementary material for: The influence of culture and cognitive reserve on the clinical presentation of behavioural-variant frontotemporal dementia
Source: J Neurol. 2023 Mar 14;270(6):3192–203. doi: 10.1007/s00415-023-11638-w (PMC10188392; doi:10.1007/s00415-023-11638-w)
Supplement: Supplementary file 1 — Supplementary file1 (DOCX 167 KB) [file 415_2023_11638_MOESM1_ESM.docx]

**Supplementary Materials**

Title: The influence of culture and cognitive reserve on the clinical presentation of behavioural-variant frontotemporal dementia

Skeggs, A^1,2^, Wei, G^1,2^, Landin-Romero, R^1,2^, Hodges, JR^2,3^, Piguet, O^1,2^ & Kumfor, F^1,2^

**Correspondence to:** Fiona Kumfor**,** 94 Mallett Street Camperdown, Sydney, NSW, Australia 2050, fiona.kumfor@sydney.edu.au

**Supplementary Table 1**

Diagnostic and neuropsychiatric features across bvFTD groups

|  | Australian  *n* = 53 | CALD-English  *n* = 36 | CALD-LOTE  *n* = 18 | Chi square | *p* | *Cramer’s V* |
| --- | --- | --- | --- | --- | --- | --- |
| **Diagnostic features** |  |  |  |  |  |  |
| Disinhibition | 30 (75.0%) | 19 (82.6%) | 13 (92.9%) | 1.94 | .369 | .17 |
| Apathy | 34 (85.0%) | 21 (91.3%) | 11 (78.6%) | 1.28 | .560 | .12 |
| Loss of Empathy | 35 (87.5%) | 17 (73.9%) | 10 (71.4%) | 2.85 | .254 | .18 |
| Preservative Behaviour | 21 (52.5%) | 11 (47.8%) | 6 (42.9%) | 0.42 | .812 | .07 |
| Hyperorality | 30 (75%) | 14 (60.9%) | 13 (92.9%) | 4.50 | .105 | .25 |
| **Neuropsychiatric features** |  |  |  |  |  |  |
| Abnormal Motor Behaviour | 23 (50.0%) | 13 (41.9%) | 3 (21.4%) | 3.53 | .170 | .20 |
| Agitation | 28 (59.6%) | 20 (66.7%) | 9 (64.3%) | 0.44 | .874 | .07 |
| Anxiety | 14 (30.4%) | 17 (56.7%) | 4 (28.6%) | 5.78 | .053 | .26 |
| Apathy | 39 (83%) | 28 (90.3%) | 12 (85.7%) | 0.85 | .716 | .10 |
| Appetite | 40 (85.1%) | 22 (73.3%) | 12 (85.7%) | 1.78 | .377 | .14 |
| Delusions | 12 (25%) | 8 (25.8%) | 2 (14.3%) | 0.71 | .714 | .09 |
| Depression | 18 (39.1) | 8 (28.6%) | 4 (30.8%) | 0.93 | .676 | .11 |
| Disinhibition | 31 (66%) | 22 (71%) | 11 (78.6%) | 0.77 | .675 | .10 |
| Elation | 25 (53.3%) | 6 (20%) | 4 (28.6%) | 9.11 | .010* | .32 |
| Hallucinations | 5 (10.6%) | 5 (16.1%) | 1 (7.1%) | 0.81 | .675 | .10 |
| Irritability | 24 (52.2%) | 15 (50%) | 10 (71.4%) | 1.91 | .390 | .15 |
| Sleep | 23 (50%) | 12 (38.7%) | 5 (38.5%) | 1.17 | .593 | .11 |

*Note*. % of cases who satisfied criteria at presentation. For diagnostic features and neuropsychiatric features data were from clinical assessments and displayed as N (%). Missing data for neuropsychiatric features: abnormal motor behaviour 16; agitation: 15; anxiety: 15; apathy: 14; appetite: 14; delusions: 14; depression: 19; disinhibition: 15; elation: 15; hallucinations: 15; irritability: 15; sleep: 15. Significant post hoc test: elation: Australia>Foreign groups.

**Supplementary Figure 1** Number of diagnostic features met at initial clinical assessment

*Note.* A chi-square test was used to examine differences in the number of diagnostic criteria fulfilled by the bvFTD groups at clinical assessment. No significant differences were found

**Supplementary Table 2** Neuropsychological test scores across groups

|  | Australian | CALD-English | CALD-LOTE | Controls | *F* | *p* | Post hoc |
| --- | --- | --- | --- | --- | --- | --- | --- |
|  | *n* = 53 | *n* = 36 | *n* = 18 | *n =* 51 |  |  |  |
| **Cognitive Function**  ACE-III | 75.33 ± 16.13 | 74.57 ± 15.54 | 74.59 ± 17.43 | 95.29 ± 3.21 | 16.10 | <.001 | All groups < Controls |
| **Attention/Working Memory** |  |  |  |  |  |  |  |
| Digit span forwards | 6.10 ± 1.31 | 6.22 ± 1.26 | 5.71 ± .77 | 7.08 ± 1.21 | 4.66 | .004 | Aus, FNE < Controls |
| Digit span backwards | 3.84 ± 1.32 | 4.03 ± 1.51 | 3.88 ± .86 | 5.49 ± 1.14 | 7.76 | <.001 | All groups < Controls |
| Trail A time (s) | 56.68 ± 43.82 | 59.15 ± 35.35 | 46.19 ± 24.19 | 32.04 ± 11.68 | 9.80 | <.001 | All groups < Controls |
| **Visuospatial Function** |  |  |  |  |  |  |  |
| RCF copy score (36) | 25.36 ± 8.14 | 27.76 ± 5.70 | 29.74 ± 4.50 | 32.79 ± 2.98 | 8.45 | <.001 | Aus, FE < Controls |
| **Episodic Memory** |  |  |  |  |  |  |  |
| RCF 3 min score (36) | 9.71 ± 8.84 | 10.05 ± 7.61 | 13.06 ± 6.22 | 18.03 ± 5.35 | 12.38 | <.001 | All groups < Controls |
| **Language**  SYDBAT |  |  |  |  |  |  |  |
| Naming (30) | 21.82 ± 5.51 | 21.39 ± 6.11 | 20.47 ± 5.81 | 27.37 ± 1.54 | 9.71 | <.001 | All groups < Controls |
| Semantic Association (30) | 24.19 ± 4.85 | 23.45 ± 5.69 | 25.40 ± 3.81 | 28.43 ± 1.23 | 8.64 | <.001 | All groups < Controls |
| **Executive Function** |  |  |  |  |  |  |  |
| Trail B time (s) | 131.45 ± 74.58 | 164.90 ± 118.6 | 129.93 ± 68.79 | 74.48 ± 26.67 | 9.42 | <.001 | All groups < Controls |
| Verbal Fluency | 22.92 ± 15.32 | 26.39 ± 16.06 | 22.44 ± 10.83 | 47.53 ± 12.10 | 14.03 | <.001 | All groups < Controls |

Data were presented as mean ± standard deviation. Sidak correction for multiple comparisons: critical *p* = .008. Abbreviations: bvFTD = behavioral variant frontotemporal dementia, CALD = culturally and linguistically diverse, CALD-LOTE = culturally and linguistically diverse – language other than English, ACE = Addenbrooke’s Cognitive Examination, RCF = Rey Complex Figure, SYDBAT = Sydney Language Battery. Missing data: ACE-III: 4 CALD-English, Digit span forwards: 3 Australian; 4 CALD-English; 1 CALD-LOTE; 2 Controls, Digit span backwards: 3 Australian; 5 CALD-English; 2 CALD-LOTE; 5 Controls, Trail A time: 3 Australian; 5 CALD-English; 2 CALD-LOTE; 5 Controls, Trail B time: 4 Australian; 6 CALD-English; 2 CALD-LOTE; 5 Controls, RCF copy score: 4 Australian; 5 CALD-English; 1 CALD-LOTE; 5 Controls, RCF 3 min score: 4 Australian; 6 CALD-English; 2 CALD-LOTE; 5 Controls, SYDBAT naming: 3 Australian; 5 CALD-English; 3 CALD-LOTE; 5 Australians, SYDBAT semantic association: 13, Verbal fluency: 5 Australians; 5 CALD-English; 2 CALD-LOTE; 8 Controls.

**Supplementary Table 3**

Voxel-based morphometry results showing regions of grey matter intensity reduction in bvFTD patient groups compared to controls

| **Regions** | **Hemisphere** | **Number of voxels** | **MNI coordinates** | | |  |
| --- | --- | --- | --- | --- | --- | --- |
|  |  |  | *X* | *Y* | *Z* | |
| **bvFTD: Australian** |  |  |  |  |  | |
| Temporal Pole, Orbitofrontal Cortex, Medial Frontal Cortex, Frontal Pole, Parahippocampal Gyrus, Amygdala, Hippocampus, Putamen, Insular Cortex, Accumbens | B | 74520 | -28 | -4 | -52 | |
| Cerebellum (Crus I, II, VIIb) | R | 6109 | 52 | -62 | -44 | |
| **bvFTD: CALD-English** |  |  |  |  |  | |
| Putamen, Amygdala, Hippocampus, Insular Cortex, Orbitofrontal Cortex, Planum Polare, Heschl's Gyrus (H1, H2), Parietal Operculum Cortex, Frontal Pole, Temporal Pole, Frontal Operculum Cortex | R | 58923 | 28 | 4 | -30 | |
| Cerebellum (Crus I, II, VIIb) | L | 2463 | -40 | -78 | -48 | |
| Cerebellum (Crus I, II, VIIb) | R | 564 | 46 | -56 | -56 | |
| **bvFTD: CALD-LOTE** |  |  |  |  |  | |
| Cerebellum (Crus I, II, VI), Temporal Fusiform Cortex (posterior division), Temporal Occipital Fusiform Cortex, Lateral Occipital Cortex (inferior division), Occipital Fusiform Cortex | R | 54920 | 42 | -56 | -58 | |
| Occipital Pole, Lateral Occipital Cortex (inferior division), Occipital Fusiform Gyrus | L | 926 | -30 | -92 | -12 | |
| Occipital Fusiform Gyrus, Occipital Pole | R | 492 | 22 | -88 | -8 | |

*Note.* Results are reported at *p* <0.05 corrected for family-wise error, with a cluster threshold of 300 contiguous voxels. CALD = culturally and linguistically diverse; CALD-LOTE = culturally and linguistically diverse – language other than English; B = bilateral; L = left; R = right; MNI = Montreal Neurological Institute.
